# The Effect of the Microalgae *Chlorella vulgaris* on the Gut Microbiota of Juvenile Nile Tilapia (*Oreochromis niloticus*) Is Feeding-Time Dependent

**DOI:** 10.3390/microorganisms11041002

**Published:** 2023-04-12

**Authors:** Zhicheng Huang, Jinyan Gao, Chunyan Peng, Jingjing Song, Zongsheng Xie, Jixin Jia, Haochen Li, Shumiao Zhao, Yunxiang Liang, Bin Gong

**Affiliations:** 1 State Key Laboratory of Agricultural Microbiology, College of Life Science and Technology, Huazhong Agricultural University, Wuhan 430070, China; 2 The Guangxi Key Laboratory of Beibu Gulf Marine Biodiversity Conservation, College of Marine Sciences, Beibu Gulf University, Qinzhou 535011, China

**Keywords:** *Chlorella vulgaris*, gut microbiota, life stage, diversity, co-occurrence network

## Abstract

*Chlorella vulgaris* is one of the most commonly used microalgae in aquaculture feeds. It contains high concentrations of various kinds of nutritional elements that are involved in the physiological regulation of aquaculture animals. However, few studies have been conducted to illustrate their influence on the gut microbiota in fish. In this work, the gut microbiota of Nile tilapia (*Oreochromis niloticus*) (average weight is 6.64 g) was analyzed by high-throughput sequencing of the 16S rRNA gene after feeding with 0.5% and 2% *C. vulgaris* additives in diets for 15 and 30 days (average water temperature was 26 °C). We found that the impact of *C. vulgaris* on the gut microbiota of Nile tilapia was feeding-time dependent. Only by feeding for 30 days (not 15 days) did the addition of 2% *C. vulgaris* to diets significantly elevate the alpha diversity (Chao1, Faith pd, Shannon, Simpson, and the number of observed species) of the gut microbiota. Similarly, *C. vulgaris* exerted a significant effect on the beta diversity (Bray–Curtis similarity) of the gut microbiota after feeding for 30 days (not 15 days). During the 15-day feeding trial, LEfSe analysis showed that *Paracoccus*, *Thiobacillus*, *Dechloromonas*, and *Desulfococcus* were enriched under 2% *C. vulgaris* treatment. During the 30-day feeding trial, *Afipia*, *Ochrobactrum*, *Polymorphum*, *Albidovulum*, *Pseudacidovorax*, and *Thiolamprovum* were more abundant in 2% *C. vulgaris-*treated fish. *C. vulgaris* promoted the interaction of gut microbiota in juvenile Nile tilapia by increasing the abundance of *Reyranella*. Moreover, during the feeding time of 15 days, the gut microbes interacted more closely than those during the feeding time of 30 days. This work will be valuable for understanding how *C. vulgaris* in diets impacts the gut microbiota in fish.

## 1. Introduction

*Chlorella vulgaris* is one of the most commonly used microalgae in aquaculture feeds [1]. It has been incorporated as an ingredient in the diets of aquaculture animals, such as prawns [2], Nile tilapia [3,4], rainbow trout [5], and African catfish [6]. *C. vulgaris* contains high concentrations of lutein, beta-carotene, astaxanthin, canthaxanthin, and chlorella growth factor (CGF) [1]. *C. vulgaris* leads to higher growth, better feed utilization, and increased digestive enzymatic activities in fish when used as a feed additive [7,8,9]. The addition of some amount of *C. vulgaris* to diets also modulated and increased the antioxidant capacity, innate immunity, and disease resistance of fish [10]. *C. vulgaris* also contains high-quality protein and lipids, and has been used at higher inclusions (10–20%) in the diet to replace fishmeal [11]. However, few studies have been conducted to illustrate the influence of *C. vulgaris* on the gut microbiota in fish.

The gut microbiota plays many critical roles in host fish [12,13]. In fish, the early larval and juvenile stages represent a critical period for growth and survival in aquaculture because larvae are vulnerable to diseases, such as infection, during this stage [14]. The gut microbiota in larval and juvenile fish is crucial for protection against pathogens and for nutrition metabolism and physiological functions [15]. Diets have been successfully used to modulate the gut microbiota and further promote the health of vertebrate animals [16,17]. With the rapid development of high-throughput sequencing, the in-depth mechanisms by which *C. vulgaris*, as a feed additive, impacts the gut microbial communities in aquaculture animals have been illustrated [1]. It was found that the abundance of beneficial bacterial taxa (Firmicutes, Bacteroidetes, and Cetobacterium) in the gut of juvenile largemouth bass (*Micropterus salmoides*) was significantly increased by feeding with *C. vulgaris* [18]. Moreover, fishmeal partially substituted with *C. vulgaris* increased the abundance of *Lactobacillus* and decreased *Escherichia coli* abundance in the gut of juvenile narrow clawed crayfish [19]. Notably, although some microalgae, such as *Schizochytrium* [20], have been studied, we still know little about how *C. vulgaris* shapes the microbial communities in the gastrointestinal (GI) tract in Nile tilapia (*Oreochromis niloticus*), especially larval and juvenile fish, until now. Moreover, the digestive systems and immune organs of larval and juvenile fish develop rapidly [14]. We hypothesized that the effect of *C. vulgaris* meal on the gut microbiota in larval and juvenile fish may be dependent on feeding time. In this work, after feeding with 0.5% and 2% *C. vulgaris* additives in diets for 15 and 30 days, the gut microbiota in Nile tilapia were analyzed by high-throughput sequencing of the 16S rRNA gene. We aimed to determine how *C. vulgaris* in diets impacts the gut microbiota in juvenile Nile tilapia at different feeding times.

## 2. Materials and Methods

### 2.1. Feed Preparation

*C. vulgaris* was obtained from the Freshwater Algae Culture Collection at the Institute of Hydrobiology (FACHB) in China. *C. vulgaris* was cultured with growth media (CaCl_2_•2H_2_O 1 g/L, FeSO_4_•7H_2_O 0.01 g/L, MgSO_4_•7H_2_O 0.2 g/L, NaNO_3_ 2 g/L, K_2_HPO_4_ 0.2 g/L, yeast extract 1 g/L, glucose 4 g/L) in 10-L sterilized bottles for one week. Afterward, *C. vulgaris* was centrifuged and air-dried at room temperature for collection. The *C. vulgaris* and other components of the diets are listed in Table 1. All the dietary components were ground, sifted (60-mesh sieve), and mixed evenly. Afterward, the mixed raw materials were pressed into pellets with a fish feed pelleting machine (SZLH250; HENGFU MACHINERY, China) using a cold pelleting process, and the screen diameter was 2 mm; the pellets were then air-dried (20 °C), and stored at 4–8 °C until use.

### 2.2. Feeding and Management

A total of 270 healthy Nile tilapia (weight: 6.64 ± 0.88 g) were selected and evenly divided into three groups (control group: fed a basal diet; 0.5% Chl group: fed a basal diet supplemented with 0.5% *C. vulgaris*; 2% Chl group: fed a basal diet supplemented with 2% *C. vulgaris*) in nine 300 L fiberglass tanks (three tanks per group). Nile tilapia were maintained in an indoor recirculating freshwater system with temperature control and aeration equipment at Beibu Gulf University (Qinzhou city, China). Water quality was tested daily and properly managed as follows: temperature, 26 ± 1.5 °C; dissolved oxygen, >6 mg L^−1^; pH, 7.2 ± 0.5; ammonia nitrogen < 0.04 mg L^−1^. Fish were fed at 2% to 3% body weight by hand twice daily (8:00–9:00 and 17:00–18:00) for 30 days.

### 2.3. DNA Extraction and 16S rRNA Gene High-Throughput Sequencing of the Gut Microbiota

At the end of the feeding trial, healthy fish with similar weights were collected from each group (six fish from each group). The fish surface was wiped and sterilized with 70% alcohol, and then the fish were placed on a clean dissecting pan. The intestinal contents of the fish were obtained with a sterilized dissecting knife, immediately frozen in liquid nitrogen, and stored in a −80 °C freezer. Then, the total DNA of the gut microbiota in Nile tilapia was extracted from 0.3 g feces (all the feces from fish guts were homogenized before use) with the GenElute™ Stool DNA Isolation Kit (Sigma–Aldrich, St. Louis, MO, USA). After assessing the quality of the DNA, the obtained DNA was used as a template for amplification of the V3–V4 region in the 16S rRNA gene with primers 341F and 909R [21]. Then, high-throughput sequencing was conducted using the Illumina HiSeq2500 platform [22] at Shanghai Majorbio Biopharm Technology Co., Ltd. (Shanghai, China). The raw data were processed with the QIIME 2 pipeline (version 2020.2) following the following procedure: trimming with StreamingTrim [23], merging with MeFiT [24], and chimeric assessment and removal with Greengenes [25]. The clean reads were used to construct amplicon sequence variants (ASVs) with DADA2 in R [26]. The taxonomic annotation of the ASVs was conducted with the SINTAX classifier in usearch (version 11.0.667) [27] against the Silva Database (version 138.1) [28]. A total of 19,439 ASVs were generated from 37 samples, with an average of 84,961 clean reads per sample.

### 2.4. Bioinformatics Analysis

The alpha diversity indices (Chao1, Shannon, Simpson, number of observed species, Faith_pd, and Pielou_e index) of gut microbes were calculated using the phyloseq package in R [29]. The beta diversity of the gut microbiota in fish was calculated using Bray–Curtis dissimilarity [30], and then the results were visualized with nonmetric dimensional scaling (NMDS) using ggplot2 in R [31]. Linear discriminant analysis effect size (LEfSe) analysis of the gut microbiota in Nile tilapia was performed on the Majorbio Cloud Platform with an LDA threshold of 3–4 (FDR < 0.01) [32]. To assess interactions among microbial communities (using operational taxonomic units (OTUs)) within the gut of Nile tilapia in two growth stages (15 days and 30 days), a cooccurrence network analysis was implemented. Networks were calculated using the online tool of the Majorbio Cloud Platform (https://cloud.majorbio.com/page/tools/, accessed on 21 December 2021) [33]. The Pearson correlation coefficient was utilized as an index to evaluate the interaction. Strong (|R| > 0.8) and significant (*p* < 0.05) interactions were used to build the co-occurrence networks. The networks were rendered using Gephi software (Version 0.9.2).

### 2.5. Statistical Analysis

One-way analysis of variance (one-way ANOVA) was used to test the differences in alpha diversity variation of gut microbiota among different groups. The statistical analyses were performed in SPSS 24.0 software [34], and *p* < 0.05 was considered a significance threshold. Analysis of similarities (ANOSIM) was used to test for variations in β-diversity in the gut microbiota between groups in R 4.1.3.

## 3. Results

### 3.1. Alpha Diversity of the Gut Microbiota

**Figure 1:**
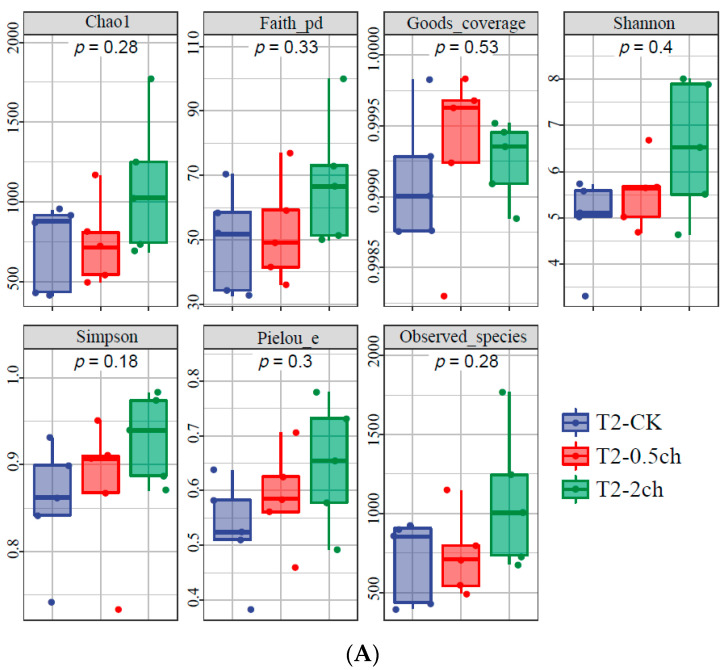

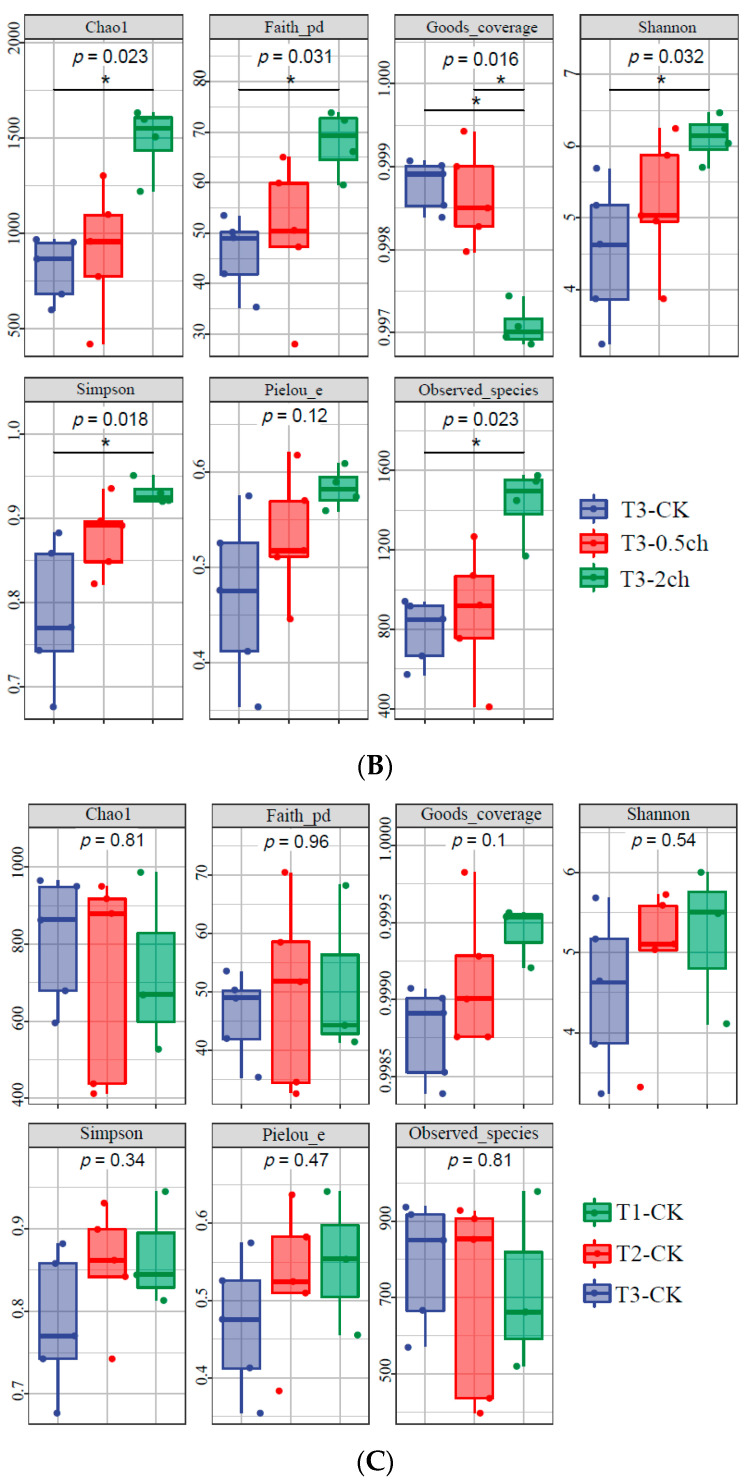
The alpha diversity of the microbial community in the gut of Nile tilapia (*Oreochromis niloticus*). (**A**). The alpha diversity of the microbiota in Nile tilapia was fed 0.5% and 2% *C. vulgaris* for 15 days. T2-CK, control group; T2-0.5ch, 0.5% *C. vulgaris*-treated group; T2-2ch, 2% *C. vulgaris*-treated group. (**B**). The alpha diversity of the microbiota in Nile tilapia fed 0.5% and 2% *C. vulgaris* for 30 days. T3-CK, control group; T3-0.5ch, 0.5% *C. vulgaris*-treated group; T3-2ch, 2% *C. vulgaris*-treated group. (**C**). The alpha diversity of the microbiota in Nile tilapia during different feeding times. T1-CK, before the feeding trial; T2-CK, fed for 15 days without *C. vulgaris*; T2-CK, fed for 30 days without *C. vulgaris*. Symbol * means *p* < 0.01.

The alpha diversity of the microbial community in the gut of Nile tilapia was detected and analyzed. The alpha diversity of the gut microbiota in Nile tilapia fed *C. vulgaris* for 15 days (Figure 1A) and 30 days (Figure 1B) was compared. During this 30-day feeding trial, 2% *C. vulgaris* significantly increased the Chao1, Faith pd, Shannon, Simpson, and the number of observed species (Figure 1B) (ANOVA, *p* < 0.05). The alpha diversity of the gut microbiota at different feeding times (0 days, 15 days, and 30 days) was compared (Figure 1C). The Shannon and Simpson indices in the gut microbiota of fish fed for 0 and 15 days were higher than those in the 30-day group.

### 3.2. NMDS Analysis of the Gut Microbiota

**Figure 2:**
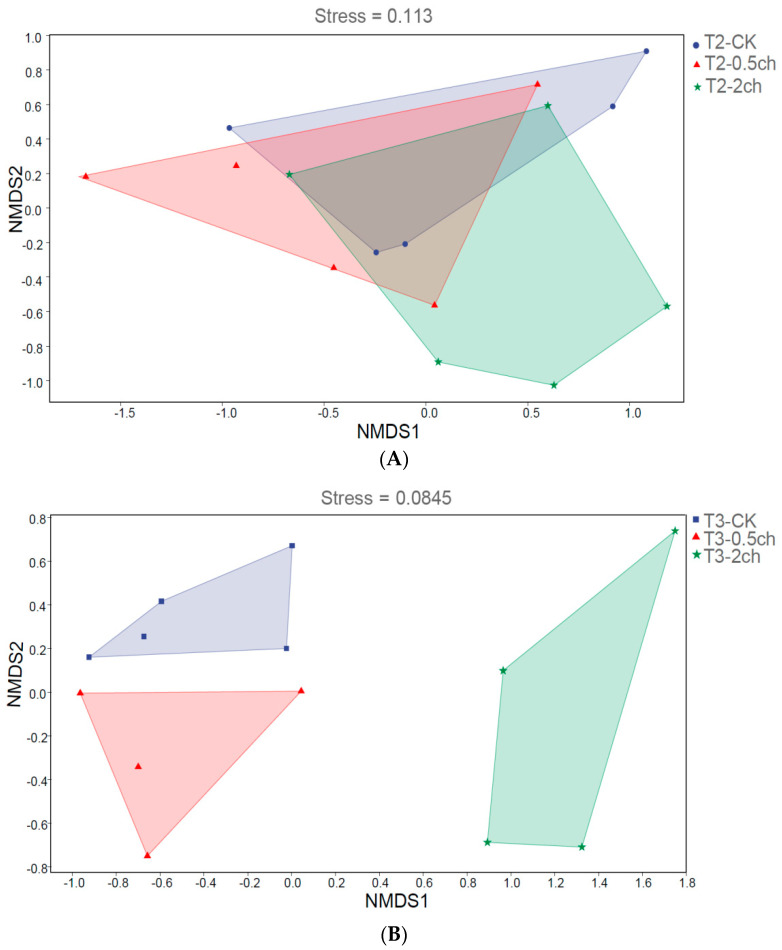

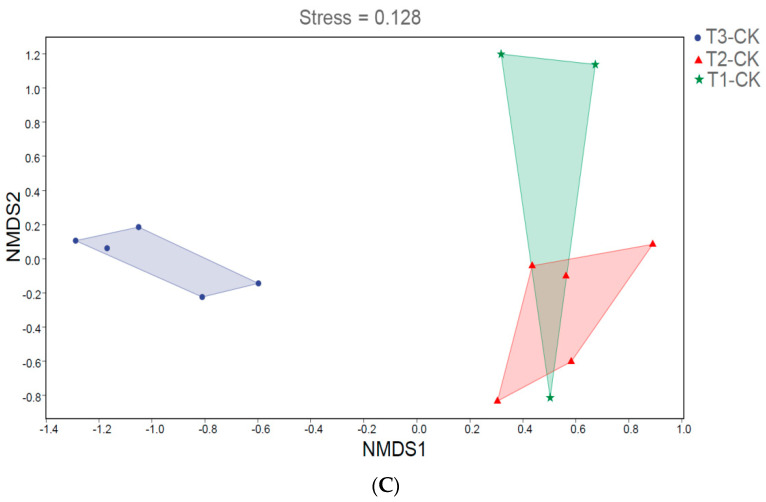
Nonmetric multidimensional scaling (NMDS) analysis of gut microbiota in Nile tilapia. (**A**). NMDS analysis of the microbiota in Nile tilapia fed 0.5% and 2% *C. vulgaris* for 15 days. T2-CK, control group; T2-0.5ch, 0.5% *C. vulgaris*-treated group; T2-2ch, 2% *C. vulgaris*-treated group. (**B**). NMDS analysis of the microbiota in Nile tilapia fed 0.5% and 2% *C. vulgaris* for 30 days. T3-CK, control group; T3-0.5ch, 0.5% *C. vulgaris*-treated group; T3-2ch, 2% *C. vulgaris*-treated group. (**C**). NMDS analysis of the microbiota in Nile tilapia during different feeding times. T1-CK, before feeding; T2-CK, fed for 15 days without *C. vulgaris*; T2-CK, fed for 30 days without *C. vulgaris*.

We analyzed the influence of 0.5% and 2% *C. vulgaris* and growth time on the beta diversity of the gut microbiota in Nile tilapia by NMDS analysis (Figure 2). During the 15-day feeding trial, we did not observe a distinct discrepancy in the microbial community among the 0.5% *C. vulgaris*-treated, 2% *C. vulgaris*-treated, and non-Chlorella-treated groups (Figure 2A). However, the gut microbiota in Nile tilapia at 0 days and 15 days was different from that at 30 days (Figure 2C). As the figure shows, the microbial communities during the 0-day and 15-day feeding trials were closely clustered, while the microbiota during the 30-day feeding trials were distributed in another cluster. During the 30-day feeding trial, the gut microbiota in the 2% *C. vulgaris*-treated, 0.5% *C. vulgaris*-treated, and non-Chlorella-treated groups were significantly separated from each other (ANOSIM, R = 0.452, *p* = 0.001) (Figure 2B).

### 3.3. LEfSe Analysis

**Figure 3:**
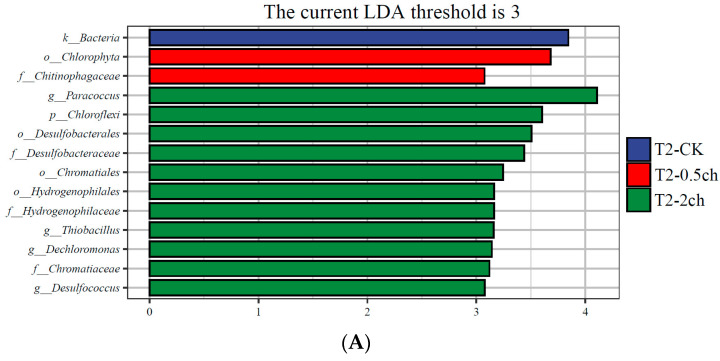

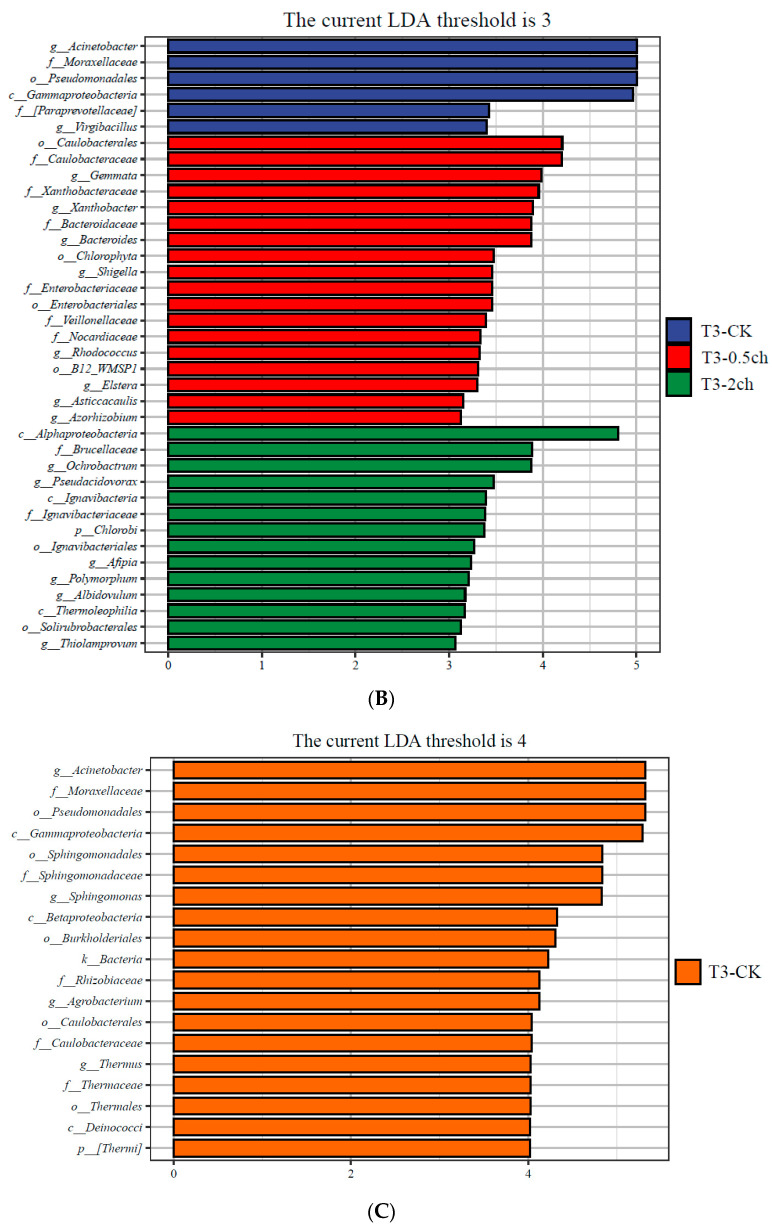
Linear discriminant analysis effect size (LEfSe) analysis of gut microbiota in Nile tilapia. (**A**) The taxa with different abundances of gut microbiota in Nile tilapia fed 0.5% and 2% *C. vulgaris* for 15 days. T2-CK, control group; T2-0.5ch, 0.5% *C. vulgaris*-treated group; T2-2ch, 2% *C. vulgaris*-treated group. (**B**) The taxa with different abundances of gut microbiota in Nile tilapia fed 0.5% and 2% *C. vulgaris* for 30 days. T3-CK, control group; T3-0.5ch, 0.5% *C. vulgaris*-treated group; T3-2ch, 2% *C. vulgaris*-treated group. (**C**) The taxa with different abundances of gut microbiota in Nile tilapia during different feeding times. T1-CK, before feeding; T2-CK, fed for 15 days without *C. vulgaris*; T3-CK, fed for 30 days without *C. vulgaris*.

LEfSe analysis was utilized to identify discriminating taxa in the gut microbiota of Nile tilapia during different feeding times (*p* < 0.05, LDA > 4) and those associated with different amounts of *C. vulgaris* in diets (*p* < 0.05, LDA > 3) (Figure 3). During a 15-day feeding trial, the gut microbiota of fish fed 2% *C. vulgaris* was represented by biomarkers such as *Paracoccus*, *Thiobacillus*, *Dechloromonas*, and *Desulfococcus*. During a 30-day feeding trial, the gut microbiota of fish fed 2% *C. vulgaris* was represented by *Afipia*, *Ochrobactrum*, *Polymorphum*, *Albidovulum*, *Pseudacidovorax*, and *Thiolamprovum*; 0.5% *C. vulgaris*-enriched taxa included *Rhodococcus*, *Bacteroides*, *Gemmata*, *Asticcacaulis*, *Azorhizobium*, *Xanthobacter*, *Elstera*, and *Shigella*. During different life stages (0 days, 15 days, and 30 days), basal diet feeding for 30 days significantly stimulated the proliferation of *Thermus*, *Agrobacterium*, *Sphingomonas*, and *Acinetobacter*.

### 3.4. Network Analysis

**Figure 4:**
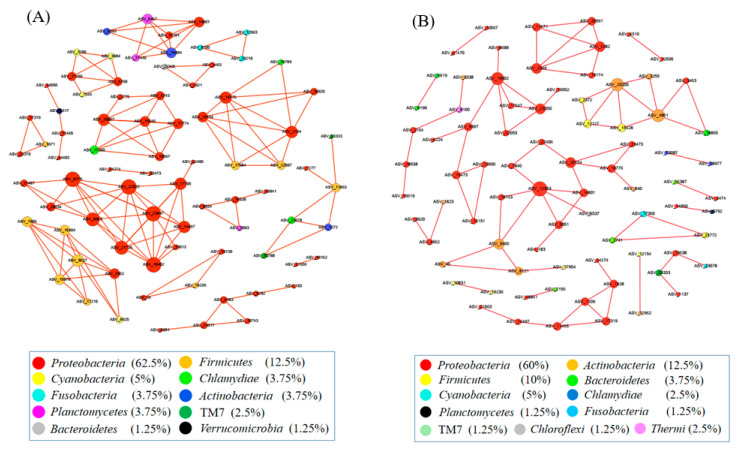
The interactions of gut microbes in Nile tilapia are illustrated by co-occurrence network analysis. (**A**). The co-occurrence network of gut microbiota in Nile tilapia fed 0%, 0.5%, and 2% *C. vulgaris* for 15 days. (**B**). The co-occurrence network of gut microbiota in Nile tilapia fed 0%, 0.5%, and 2% *C. vulgaris* for 30 days. The different colored circles indicate some ASVs in the network annotated at the phylum level.

**Figure 5:**
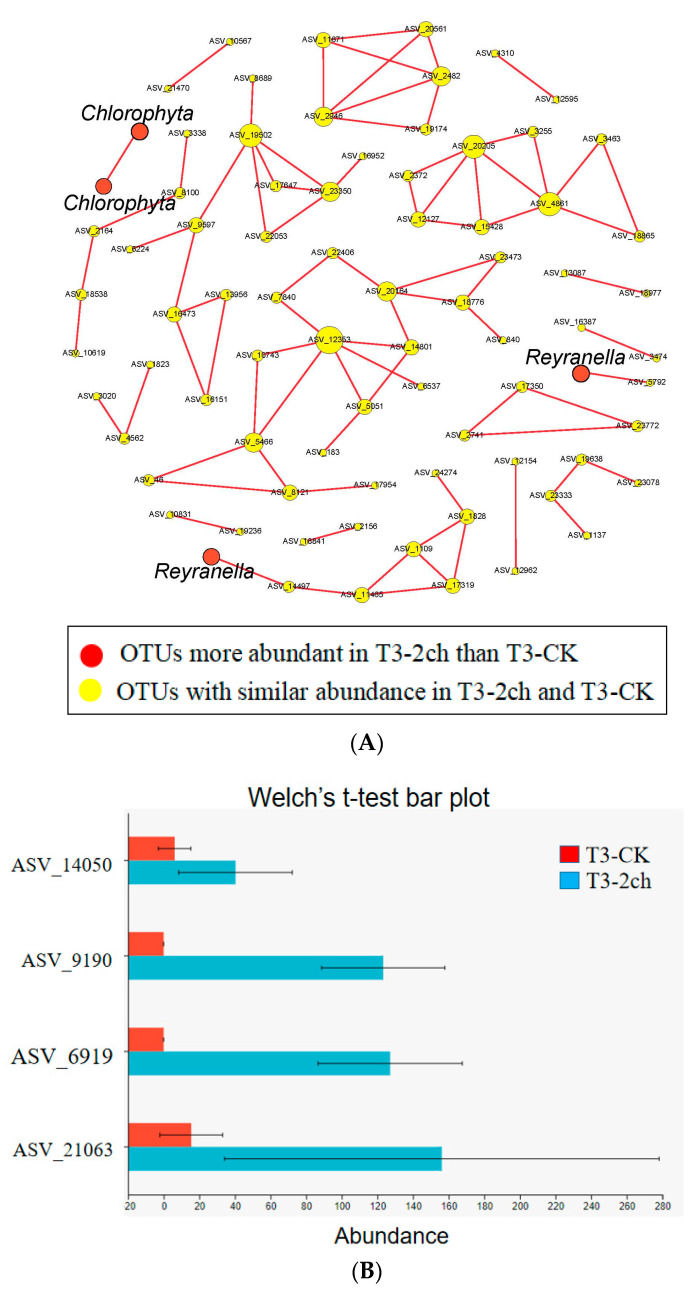
Some ASVs in the co-occurrence network were especially enriched in 2% *C. vulgaris*-treated Nile tilapia at the life stage of 30 days. (**A**) The red nodes indicate ASVs enriched in 2% *C. vulgaris*-treated fish; (**B**) The ASVs with higher abundance in 2% *C. vulgaris*-treated fish were detected by Welch’s *t*-test (*p* < 0.05).

The co-occurrence network among ASVs was constructed using random matrix theory (RMT) (Figure 4). During the growth time of 15 days, a total of 80 ASVs (nodes) and 134 correlation links (edges) were rendered in the network of gut microbiota (R > 0.80, *p*-value > 0.05) (Netwoek_15 days_, Figure 4A). During the growth time of 30 days, a total of 80 ASVs (nodes) and 82 correlation links (edges) were rendered (Netwoek_30 days_, R > 0.80, *p*-value > 0.05) (Figure 4B). Eight and five large modules, which possessed more than five nodes in each module, were recovered from Network_15 days_ and Network_30 days_, respectively. In both co-occurrence networks above, *Proteobacteria* was the most predominant (Figure 4). Four ASVs in the co-occurrence network, named ASV_9190, ASV_6919, ASV_21063, and ASV_14050, were more abundant in 2% *C. vulgaris*-treated Nile tilapia at a feeding time of 30 days (Welch’s *t*-test, *p* < 0.05) (Figure 5). ASV_9190 and ASV_6919 were annotated as unidentified_*Chlorophyta*, while ASV_21063 and ASV_14050 were annotated as *Reyranella_massiliensis*.

## 4. Discussion

Gut microbes in fish influence various host functions, including nutrition, digestion, development, disease resistance, and immunity [35]. Studies of the gut microbiota are beneficial for developing effective strategies to promote host health and improve growth [36]. Host phylogeny and diet may significantly influence gut microbial community diversity in fish [37]. Among them, the effect of diets on the microbial community present in the fish gut has recently attracted increasing attention [38]. Changes in dietary nutrients and ingredients have been applied to evaluate their distinct impact on the gut microbial community of fish [39,40,41]. The impact of different diets on the alpha and beta diversity of the gut microbiota in fish varies [38,42,43,44]. Moreover, the enzyme activity and physiological structure of the GI tract in fish may change during ontogenesis [45]. Therefore, when we evaluate the influence of diets on the gut microbial community in fish, we should consider the influence of different lifetimes. To date, we know little about this effect, and it is urgent to conduct in-depth investigations.

In this study, we found that the impact of *C. vulgaris* on gut microbiota diversity in fish is feeding-time dependent. First, the impact of *C. vulgaris* on the alpha diversity of the gut microbiota in Nile tilapia was correlated with feeding time. As the study illustrated, only by feeding for 30 days (not 15 days) did the addition of 2% *C. vulgaris* to diets significantly elevate the alpha diversity of gut microbiota. Second, the influence of *C. vulgaris* on the beta diversity of fish gut microbiota was feeding-time dependent as well. After feeding with *C. vulgaris* for 30 days, 0.5% and 2% of *C. vulgaris* exerted a significant effect on the beta diversity of the fish gut microbiota. However, the 15-day feeding trial did not significantly change the bacterial community structure from that observed during the initiation stage of feeding. The impact of *C. vulgaris* on the beta diversity of the gut microbiota in large fish was stronger than that in small fish. This is the first report that the impact of *C. vulgaris* on the diversity of gut microbiota is dependent on the growth time in fish.

Recently, many studies have reported that diet could strongly impact the GI microbiota in fish [46,47,48,49,50]. *Chlorella* has been widely used as a direct feed or an ingredient normally incorporated in diets for different stages of fish or prawn culturing [51]. The addition of *C. vulgaris* to fish diets significantly improved immunity [4,50], disease resistance [52], and growth rate [6,53,54]. However, the effect of *C. vulgaris* dietary supplementation on the fish intestinal microbiome has seldom been studied previously. Zhang et al. noted that 50% replacement of fishmeal with *Chlorella* did not influence species richness, Shannon diversity, or the community structure of the gut microbiota. However, high levels of fishmeal replacement (between 75 and 100%) significantly induced intestinal community disturbance and diversity loss in largemouth bass [55]. The effect of *C. vulgaris* on the gut microbiota depended on a simulated in vitro digestion process [56]. In summary, no study has been conducted on the impact of *C. vulgaris* on the fish gut microbiota during different feeding times.

The impact of *C. vulgaris* on the diversity of the gut microbiota in fish may correlate with the digestible feature of *C. vulgaris* in the GI tract of Nile tilapia. The cell wall of *C. vulgaris* is rigid, and it restricts the access of digestive enzymes in the GI tract to intracellular components for proper digestion and assimilation [1]. Moreover, the morphological and cellular characteristics of the GI tract in fish change during the first month of life [57]. Young larval fish cannot digest dietary components in a manner exactly analogous to that of juveniles [57]. A similar study found that the apparent digestibility of crude fiber increased with pig age, which was correlated with the levels of gut microbes (such as *Anaeroplasma*, *Campylobacter*, and *Clostridium*) in pigs at different ages [58]. However, the digestibility of *C. vulgaris* in Nile tilapia at different feeding times have seldom been investigated. We still know little about whether the changing gut microbiota during different feeding times of Nile tilapia is the driving force of the degradation and assimilation of *C. vulgaris* or whether a switch in intestinal physiology improves the degradation and digestion of *C. vulgaris* and then modulates gut microbes.

The use of precise control over dietary intake, especially nondigestible carbohydrates, to modulate gut microbes and improve health has recently attracted great attention [59]. The consumption of nondigestible or food with limited digestibility, especially nondigestible carbohydrates, could manipulate and modulate the gut microbial composition and is beneficial for human health [60,61]. In this work, LEfSe analysis showed that some biomarkers, such as *Paracoccus*, *Thiobacillus*, *Dechloromonas*, and *Desulfococcus*, were enriched in the gut microbiota of fish under 2% *C. vulgaris* treatment during a 15-day feeding trial. *Paracoccus*, *Thiobacillus*, *Dechloromonas*, and *Desulfococcus* are chemolithotrophic bacteria. *Thiobacillus and Desulfococcus* are believed to play major roles in sulfur oxidation and sulfate reduction, respectively. *Paracoccus* has been reported as the core microbiome in the gut of prawns [62] and shrimp [63]. Moreover, *Paracoccus* promoted growth and enhanced intestinal innate immunity in sea cucumbers [64]. Notably, the abundance of *Paracoccus* increased in the gut microbiota of fish under 2% *C. vulgaris* treatment during the 15-day feeding trial, which exhibited the beneficial effects of *C. vulgaris* on promoting the growth of probiotics in the fish gut. Traditional Chinese medicine (TCM) is believed to be beneficial for enhancing fish immune functions and improving intestinal health conditions [65]. *Dechloromonas* was found to be significantly more abundant in the intestinal microbiota of gibel carp fed TCM than in nontreated fish [66]. This finding further illustrates that *C. vulgaris* is able to promote the growth of gut bacteria beneficial for the health of fish. Some taxa, such as *Polymorphum*, were more abundant in 2% *C. vulgaris*-treated fish during the 30-day feeding trial. *Polymorphum* was detected in the gut of *Litopenaeus vannamei* fed the probiotic *Lactobacillus pentosus* but not in the control group [67]. However, the function of some other taxa, including *Afipia*, *Ochrobactrum*, *Albidovulum*, *Pseudacidovorax*, and *Thiolamprovum*, in the fish gut has not been reported previously. Therefore, a profound investigation should be conducted to illustrate their effect on the fish gut.

The co-occurrence network analysis showed some interesting results on how *C. vulgaris* impacted the gut microbiota of Nile tilapia. First, this finding indicated that the addition of *C. vulgaris* to diets did not significantly impact the interaction of microbial communities in the gut of Nile tilapia during the feeding time of 15 days. One reason for this may be that the rigidity of the cell wall restricts the utilization of *C. vulgaris* [1], and another reason could be that the abundant extracellular polysaccharides in *C. vulgaris* may restrict its absorption by fish [68]. However, *Reyranella*, which was more abundant in 0.5% or 2% *C. vulgaris*-treated Nile tilapia during the feeding time of 30 days, formed an interaction network with *Rhodobacter*, *Rhizobiales*, *Hyphomicrobium*, and *Sphingomonas*. This means that *C. vulgaris* may promote the interaction of gut microbiota by increasing the abundance of *Reyranella. Reyranella*, *Rhodobacter*, *Hyphomicrobium*, and *Sphingomonas* have been commonly found in Nile tilapia and other fish guts [69,70,71], and their functions in the gut have not been clearly illustrated until now. Second, in this work, more correlation links and modules were presented in the network during the feeding time of 15 days than at 30 days in the fish gut. This finding indicated that feeding time significantly influenced the interaction of microbes in the fish gut. More precisely, the microbes in the fish gut during the feeding time of 15 days interact more closely than those during the feeding time of 30 days. This finding could be explained by the fact that the digestive function of the fish gut during the growth time of 30 days was more developed than that of 15 days. Therefore, the digestion of food in the GI tract of fish during a feeding time of 15 days requires more cooperation of microbes, which collaborate and form a closer interaction network.

## 5. Conclusions

The impact of *C. vulgaris* on the gut microbiota of juvenile Nile tilapia is feeding time dependent. Feeding for 30 days, but not 15 days, with 2% *C. vulgaris* significantly elevated the alpha diversity of the gut microbiota, and treatment with 0.5% and 2% *C. vulgaris* significantly affected the beta diversity of the gut microbiota. During the 15-day feeding trial, *Paracoccus*, *Thiobacillus*, *Dechloromonas*, and *Desulfococcus* were enriched in the 2% *C. vulgaris* group. During the 30-day feeding trial, *Afipia*, *Ochrobactrum*, *Polymorphum*, *Albidovulum*, *Pseudacidovorax*, and *Thiolamprovum* were more abundant in 2% *C. vulgaris-*treated fish. The gut microbes during the feeding time of 15 days interacted more closely than those during the feeding time of 30 days. *C. vulgaris* promotes the interaction of gut microbiota in juvenile Nile tilapia by increasing the abundance of *Reyranella* and further promotes the interaction of *Rhodobacter*, *Rhizobiales*, *Hyphomicrobium*, and *Sphingomonas.* This work will be valuable for understanding how *C. vulgaris* in diets impacts the gut microbiota of fish.

## Figures and Tables

**Table 1:** Formulation and proximate composition of the experimental diets (%).

Ingredient	Control	0.5% Chl	2% Chl
Fish meal	20.0	20.0	20.0
Corn flour	14.0	14.0	14.0
Soybean meal	30.0	29.5	28.0
Peanut bran	10.0	10.0	10.0
Rice bran	2.5	2.5	2.5
Flour	12.3	12.3	12.3
Shrimp shell	5.0	5.0	5.0
Lecithin oil	0.5	0.5	0.5
Soybean oil	3.0	3.0	3.0
Calcium dihydrogen phosphate	2.0	2.0	2.0
Alpha starch	0.2	0.2	0.2
Vitamin and mineral premix	0.5	0.5	0.5
*C. vulgaris*	0.0	0.5	2.0
Proximate composition (% dry matter)			
Moisture	10.6	10.9	10.3
Crude protein	28.5	30.7	31.2
Crude lipid	8.1	7.4	7.3
Ash	10.4	11.3	11.1
Crude fiber	8.78	8.86	8.91
Gross energy	19.87	20.03	20.32

## Data Availability

The high-throughput sequencing data from this research has been uploaded to GenBank with the BioProject accession number PRJNA924963, and the data will be released in 24 November 2024.
